# Crystal structure of caesium dimethyl-*N*-benzoyl­amido­phosphate monohydrate

**DOI:** 10.1107/S2056989022012166

**Published:** 2023-01-06

**Authors:** Nataliia S. Kariaka, Viktoriya V. Dyakonenko, Svitlana V. Shishkina, Tatiana Yu. Sliva, Vladimir M. Amirkhanov

**Affiliations:** a Department of Chemistry, Kyiv National Taras Shevchenko University, Volodymyrska, str. 64, 01601 Kyiv, Ukraine; b SSI "Institute for Single Crystals", National Academy of Sciences of Ukraine, Nauky ave. 60, 61001 Kharkiv, Ukraine; Tulane University, USA

**Keywords:** crystal structure, carbacyl­amido­phosphate, caesium

## Abstract

The caesium salt of dimethyl-*N*-benzoyl­amido­phosphate, namely, aqua­[di­meth­yl (*N*-benzoyl­amido-κ*O*)phospho­nato-κ*O*]caesium, [Cs(C_9_H_11_NO_4_P)(H_2_O)] or Cs*L*·H_2_O, is reported. The compound crystallizes in the monoclinic crystal system in the *P*2_1_/*c* space group and forms a mono-periodic polymeric structure due to the bridging function of the dimethyl-*N*-benzoyl­amido­phosphate anions towards the caesium cations.

## Abstract 2

The crystal structure of the caesium salt of dimethyl-*N*-benzoyl­amido­phosphate is reported and discussed.

## 1. Chemical context

The carbacyl­amido­phosphates {CAPh, compounds of general formula [*R*C(O)N(H)P(O)*R*′_2_]}, first introduced by Alexandr Kirsanov in the 1960s, have now become an intensively investigated class of ligands (Amirkhanov *et al.*, 2014 ▸). The structures of the alkali metal salts of CAPh anions, important starting reagents for the synthesis of their transition-metal complexes, have been poorly studied to date. The sodium and potassium salts with 2,2,2-tri­chloro-*N*-(di­morpholino­phosphor­yl)acetamide (HCAPh^1^) contain ligated water mol­ecules and have general formulae Na_2_CAPh^1^_2_·2H_2_O and KCAPh^1^·H_2_O, respectively (Litsis *et al.*, 2010 ▸, 2016 ▸). The sodium salt of dimethyl-*N*-benzoyl­amido­phosphate NaCAPh^2^ (Kariaka *et al.*, 2019 ▸) and the alkali salts of dimethyl-*N*-trichloracetyl­amido­phosphate NaCAPh^3^, RbCAPh^3^ (Trush *et al.*, 2005 ▸) crystallize in a solvent-free form. In all of these compounds the CAPh ligands are coordinated to the metal ions in a bidentate manner (*via* the oxygen atoms of the phosphoryl and carbonyl groups) with the formation of six-membered chelate metallocycles. In addition, the phosphoryl or the carbonyl oxygen atom or both usually bridge the cations. Caesium salts of CAPhs have not been reported to date and are of inter­est as possible dopants in oxide film materials for the improvement of their electric and electron functional characteristics (Vikulova *et al.*, 2013 ▸). Because of this, an actual task is the search for caesium compounds satisfying metal–organic chemical vapor deposition requirements. The combination of caesium ions with bulky organic ligands may result in compounds with mol­ecular crystal structures that possess sufficient volatility. Thus, crystal-structure investigations of caesium salts of CAPh anions are of high inter­est. Herein, we present the crystal structure of the caesium salt of dimethyl-*N*-benzoyl­amido­phosphate. 
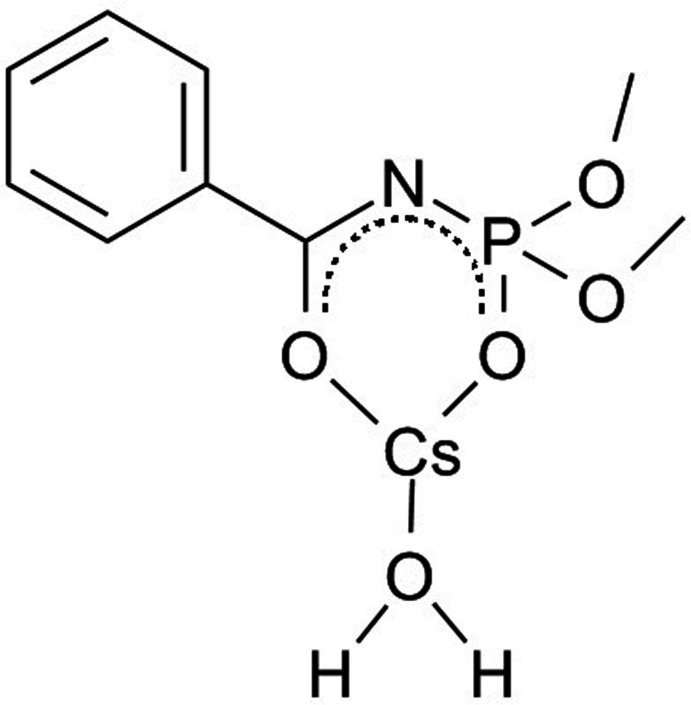


## 2. Structural commentary

**Figure 1:**
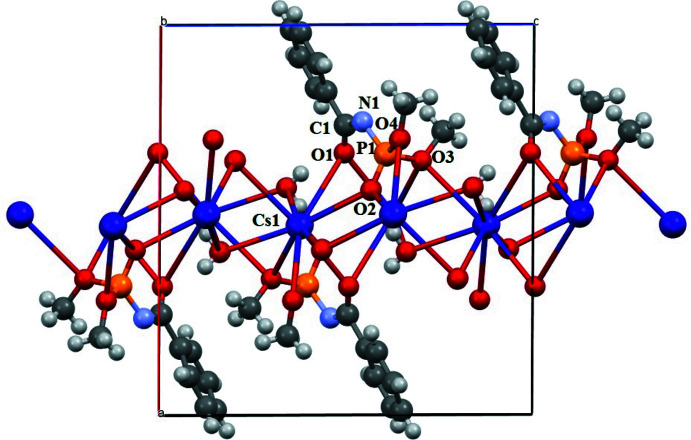
Polymeric chain of the title compound extending along the [001] crystallographic direction.

Similar to the sodium salt of dimethyl-*N*-benzoyl­amido­phosphate (Kariaka *et al.*, 2019 ▸) the title compound crystallizes in the monoclinic crystal system in the *P*2_1_/*c* space group and forms a 1D-polymeric structure (Fig. 1 ▸).

**Figure 2:**
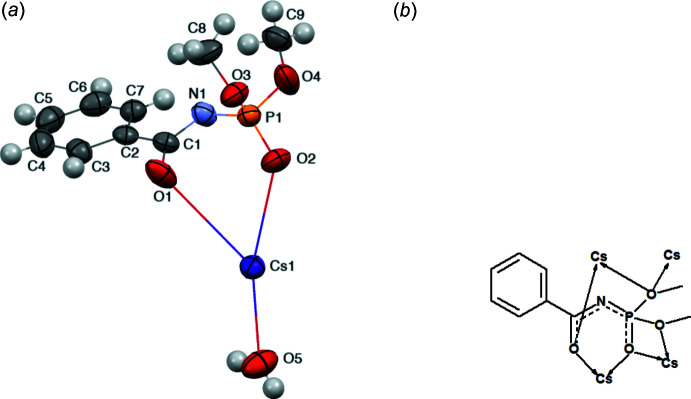
Representation of (*a*) the asymmetric unit of the title compound and (*b*) the coordination mode of *L* ^−^.

The asymmetric unit contains the Cs^+^ and CAPh^−^ ions and a water mol­ecule (Fig. 2 ▸ *a*). The oxygen atoms of the carbonyl and phosphoryl groups of the dimethyl-*N*-benzoyl­amido­phosphate anions act as μ_2_-bridges between Cs^+^ cations (Fig. 1 ▸). Additionally, both of the meth­oxy groups are bound to the Cs^+^ and one of them also acts as a μ_2_-bridge. Thus, one CAPh^−^ anion is bound to four Cs^+^ cations (Fig. 2 ▸ *b*), and each Cs^+^ cation links four ligand anions. Additionally, a water mol­ecule acts as a μ_2_-bridge between two Cs^+^ cations.

The Cs^+^ ion contacts nine oxygen atoms. It is involved in the six-membered Cs1–O1–C1–N1–P1–O2 ring with one ligand by bonding with the oxygen atoms of the carbonyl and phosphoryl groups, in the four-membered Cs1–O2–P1–O4 ring with another CAPh ligand by bonding with the oxygen atoms of the phosphoryl and meth­oxy groups and in the six-membered Cs1–O1–C1–N1–P1–O3 ring with the third ligand by bonding with the μ_2_-oxygen atoms of the carbonyl and meth­oxy groups. In addition, the Cs^+^ ion contacts with the μ_2_-O3 atom of the fourth neighboring CAPh as well as with two mol­ecules of water (Fig. 1 ▸). The six-membered chelate Cs1–O1–C1–N1–P1–O2 ring is not planar with the P1, N1 and C1 atoms deviating from the plane created through Cs1, O1 and O2 atoms by 0.471 (3), 1.403 (4) and 1.039 (4) Å, respectively. The O1—C1—N1—P1 and C1—N1—P1—O2 torsion angles are −2.4 (5) and 56.0 (3)° respectively. The six-membered Cs1–O1–C1–N1–P1–O3 ring is also not planar with the P1, N1 and C1 atoms deviating from the plane created through Cs1, O1 and O3 atoms by 0.942 (4), 0.139 (5) and 0.240 (3) Å, respectively. The C1—N1—P1—O3 torsion angle is −69.3 (3)°. The shortest Cs—O distance in the title compound (Table 1 ▸) is 3.072 (2) Å, which is comparable with the sum of the O^2−^ and Cs^+^ ionic radii (3.07 Å), so the majority of the Cs—O contacts might be considered as a mainly ionic type of bond. The Cs1—O1 distance is the longest (Table 1 ▸) and longer than the typical Cs—O bonds in crystalline solids (Leclaire *et al.*, 2008 ▸).

The average values of the C=O and P=O bond lengths in the title compound are increased as compared with H*L* [*d*(C—O)_H*L*_ = 1.219 (6) Å, *d*(P—O)_H*L*_ = 1.461 (4) Å] and the C—N and P—N bond lengths are decreased [*d*(C—N)_H*L*_ = 1.393 (7) Å, *d*(P—N)_H*L*_ = 1.667 (5) Å; Mizrahi & Modro, 1982 ▸]. Such changes are consistent with the deprotonation of H*L*.

## 3. Supra­molecular features

Few inter­molecular contacts are observed in the crystal structure of the title compound. There are O—H⋯O hydrogen bonds between the water mol­ecule and the carbonyl and phosphoryl oxygen atoms of the dimethyl-*N*-benzoyl­amido­phosphate anion (Table 2 ▸). In addition, the water mol­ecule participates in a C8—H8*C*⋯O5 contact with the hydrogen atom of the meth­oxy group of the CAPh ligand. The H8*C*⋯O5 distance is 2.56 Å. There are no inter­molecular contacts between the CAPh ligands in the crystal structure of the title compound.

## 4. Hirshfeld surface analysis and finger print plots

**Figure 3:**
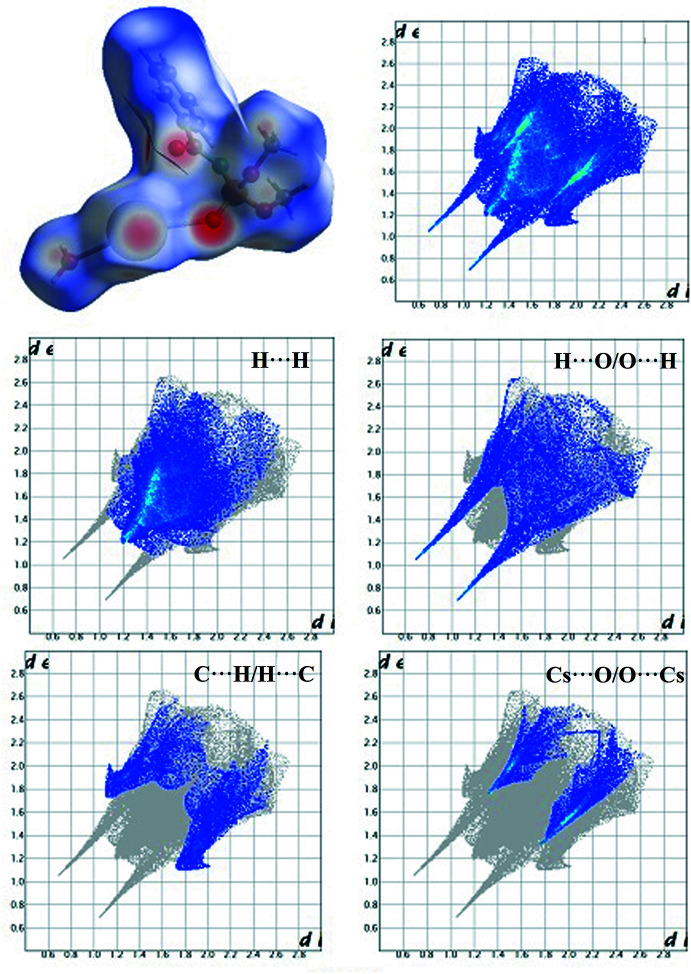
The Hirshfeld surface mapped over *d*_norm_ and two-dimensional fingerprint plots for the H⋯H (42.2%), H⋯O/O⋯H (19.3%), C⋯H/H⋯C (14.3%) and Cs⋯O/O⋯Cs (12.9%) inter­actions for the asymmetric unit of the title compound

For visualization of the inter­molecular inter­actions in the crystal structure for the asymmetric unit of the title compound, the Hirshfeld surface (Fig. 3 ▸) and its corresponding two-dimensional fingerprint plots (Spackman & Jayatilaka, 2009 ▸) were calculated using *CrystalExplorer17* (Turner *et al.*, 2017 ▸).

The dark-red spots on the surface, which correspond to the strongest contacts in the crystal structure of the title compound, are observed for the H⋯O/O⋯H hydrogen bonds between hydrogen atoms of the water mol­ecule and the oxygen atoms of the carbonyl and phosphoryl groups of the CAPh. The lighter red spots observed near the Cs^+^ cation and the meth­oxy groups correspond to Cs⋯O/O⋯Cs bonds, which are involved in Cs⋯O contacts and H⋯O contacts with the water mol­ecule. There are no red spots on the Hirshfeld surface near the phenyl ring.

The derived fingerprint plots show that H⋯H contacts make the largest contribution to the Hirshfeld surface (42.2%) and the shortest of them are at *d*_i_ = *d*_e_ = 1.2 Å. The second largest contribution (19.3%) comes from the H⋯O/O⋯H contacts, which are the shortest in the title compound (*d*_i_ + *d*_e_ = 1.75 Å). The C⋯H/H⋯C and Cs⋯O/O⋯Cs inter­actions make similar contributions to the surface at 14.3% and 12.9%, respectively. The shortest C⋯H/H⋯C contacts are at *d*_i_ + *d*_e_ = 2.8 Å. The shortest Cs⋯O/O⋯Cs contacts are at *d*_i_ + *d*_e_ = 3.07 Å. Among the inter­actions making the smallest contribution to the Hirshfeld surface of the title compound are the O⋯O, C⋯C, Cs⋯H and N⋯H inter­actions.

## 5. Database survey

A search of the Cambridge Structural Database (CSD, Version 5.42, update of November 2020; Groom *et al.*, 2016 ▸) for alkali metal salts of carbacyl­amido­phosphates yielded ten hits. Six of them are sodium salts, three are potassium salts and one is a rubidium salt. No CAPh-based caesium salts have been reported to date. In all these reported salts, the carbacyl­amido­phosphates are coordinated to the alkali metals in a bidentate chelating manner *via* the oxygen atoms of the phosphoryl and carbonyl groups. Additionally, in the majority of these salts, the phosphoryl or the carbonyl oxygen atom or both function as μ_2_-bridges. In the alkali metal salts of CAPhs that contain meth­oxy groups, one of the latter is involved in contacts with the metal. In alkali metal salts of CAPhs that contain the CCl_3_ group, the latter can be also involved in the metal binding. Some CAPh-based salts also contain such additional ligands as water mol­ecules, coordinated to the metal in a μ_2_-bridging manner, or crown ethers.

## 6. Synthesis and crystallization

Cs*L*·H_2_O was obtained by a neutralization reaction between H*L* (0.458 g, 2 mmol) and caesium carbonate (0.326 g, 1 mmol) solutions in aqueous *2*-propanol (1:3). Yield: 0.664 g, 88%, m.p. 353 K. IR (KBr): *ν*_max_ = 3408 [ν(OH)], 1591 [ν(CC)], 1535 [ν(CO)], 1378 [ν(CN)], 1205 [ν(PO)], 1076, 1038, 928 [ν(PN)], 838, 800, 734, 710, 540, 502,466, 452 cm^−1^. The low-frequency shift of *ν*(P=O) and *ν*(C=O) bands in the IR spectrum of Cs*L*·H_2_O with respect to H*L* **(**Δ*ν*_H*L*_(P=O) ∼37cm^−1^, Δ*ν*_H*L*_(C=O) ∼147cm^−1^] is typical for bidentate coordination of dimethyl-*N*-benzoyl­amido­phosphate. ^1^H NMR (DMSO-*d*_6_): *δ* = 3.24 (*s*, H_2_O), 3.54 [*d*, 6H, (OCH_3_)_2_], 7.27 (*t*, 3H, Ph), 8.04 (*d*, 2H, Ph). ^31^P NMR (acetone): *δ* = 15.2 (*s*).

## 7. Refinement

Crystal data, data collection and structure refinement details are summarized in Table 3 ▸. C-bound H atoms were positioned geometrically and refined as riding [C—H = 0.93–0.96 Å, *U*_iso_(H) = 1.2–1.5*U*_eq_(C). O-bound H atoms were refined with the restraints O5—H5*A* = O5—H5*B* = 0.84±0.01 Å and H5*A*⋯H5*B* = 1.62±0.02 Å with *U*_iso_(H) = 1.5*U*_eq_(O).

## Supplementary Material

Crystal structure: contains datablock(s) global, I. DOI: 10.1107/S2056989022012166/mw2194sup1.cif

Structure factors: contains datablock(s) I. DOI: 10.1107/S2056989022012166/mw2194Isup2.hkl

CCDC reference: 2232690

Additional supporting information:  crystallographic information; 3D view; checkCIF report

## Figures and Tables

**Table 1:** Selected geometric parameters (Å, °)

Cs1—O1^i^	3.086 (2)	Cs1—O5	3.112 (3)
Cs1—O1	3.631 (3)	Cs1—O5^iv^	3.418 (4)
Cs1—O2^ii^	3.206 (3)	P1—O2	1.468 (2)
Cs1—O2	3.072 (2)	P1—N1	1.597 (3)
Cs1—O3^iii^	3.431 (2)	O1—C1	1.247 (3)
Cs1—O3^i^	3.507 (3)	N1—C1	1.325 (4)
Cs1—O4^ii^	3.310 (2)		
			
O2—Cs1—O1	56.40 (6)	P1—O2—Cs1	131.74 (14)
O2—P1—N1	122.26 (14)	C1—N1—P1	121.5 (2)
C1—O1—Cs1	109.9 (2)	O1—C1—N1	126.3 (3)

Symmetry codes: (i) 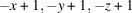 ; (ii) 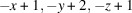 ; (iii) 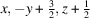 ; (iv) 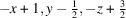 .

**Table 2:** Hydrogen-bond geometry (Å, °)

*D*—H⋯*A*	*D*—H	H⋯*A*	*D*⋯*A*	*D*—H⋯*A*
O5—H5*A*⋯O1^v^	0.85 (1)	2.05 (3)	2.785 (3)	143 (4)
O5—H5*B*⋯O2^iii^	0.86 (1)	1.87 (1)	2.721 (4)	170 (4)

Symmetry codes: (iii) 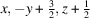 ; (v) 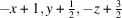 .

**Table 3:** Experimental details

Crystal data	
Chemical formula	[Cs(C_9_H_11_NO_4_P)(H_2_O)]
*M*_r_	379.08
Crystal system, space group	Monoclinic, *P*2_1_/*c*
Temperature (K)	294
*a*, *b*, *c* (Å)	14.3676 (4), 6.8089 (2), 13.7336 (3)
β (°)	90.549 (2)
*V* (Å^3^)	1343.46 (6)
*Z*	4
Radiation type	Mo *K*α
μ (mm^−1^)	2.88
Crystal size (mm)	0.5 × 0.3 × 0.2

Data collection	
Diffractometer	Xcalibur, Sapphire3
Absorption correction	Multi-scan (*CrysAlis PRO*; Agilent, 2014 ▸)
*T*_min_, *T*_max_	0.505, 1.000
No. of measured, independent and observed [*I* > 2σ(*I*)] reflections	13393, 3918, 3169
*R*_int_	0.032
(sin θ/λ)_max_ (Å^−1^)	0.703

Refinement	
*R*[*F* ^2^ > 2σ(*F* ^2^)], *wR*(*F* ^2^), *S*	0.033, 0.076, 1.03
No. of reflections	3918
No. of parameters	162
No. of restraints	3
H-atom treatment	H atoms treated by a mixture of independent and constrained refinement
Δρ_max_, Δρ_min_ (e Å^−3^)	0.52, −0.79

Computer programs: *CrysAlis PRO* (Agilent, 2014 ▸), *SHELXT2014/4* (Sheldrick, 2015*a* ▸), *SHELXL2019/2* (Sheldrick, 2015*b* ▸) and *OLEX2* (Dolomanov *et al.*, 2009 ▸).

## supplementary crystallographic information

### Crystal data

**Table:**

[Cs(C_9_H_11_NO_4_P)(H_2_O)]	*F*(000) = 736
*M**_r_* = 379.08	*D*_x_ = 1.874 Mg m^−^^3^
Monoclinic, *P*2_1_/*c*	Mo *K*α radiation, λ = 0.71073 Å
*a* = 14.3676 (4) Å	Cell parameters from 3272 reflections
*b* = 6.8089 (2) Å	θ = 3.6–28.2°
*c* = 13.7336 (3) Å	µ = 2.88 mm^−^^1^
β = 90.549 (2)°	*T* = 294 K
*V* = 1343.46 (6) Å^3^	Block, colourless
*Z* = 4	0.5 × 0.3 × 0.2 mm

### Data collection

**Table:**

Xcalibur, Sapphire3 diffractometer	3169 reflections with *I* > 2σ(*I*)
Detector resolution: 16.1827 pixels mm^-1^	*R*_int_ = 0.032
ω scans	θ_max_ = 30.0°, θ_min_ = 3.3°
Absorption correction: multi-scan (CrysAlisPro; Agilent, 2014)	*h* = −19→20
*T*_min_ = 0.505, *T*_max_ = 1.000	*k* = −9→9
13393 measured reflections	*l* = −18→19
3918 independent reflections	

### Refinement

**Table:**

Refinement on *F*^2^	Primary atom site location: difference Fourier map
Least-squares matrix: full	Hydrogen site location: mixed
*R*[*F*^2^ > 2σ(*F*^2^)] = 0.033	H atoms treated by a mixture of independent and constrained refinement
*wR*(*F*^2^) = 0.076	*w* = 1/[σ^2^(*F*_o_^2^) + (0.0307*P*)^2^ + 0.4727*P*] where *P* = (*F*_o_^2^ + 2*F*_c_^2^)/3
*S* = 1.03	(Δ/σ)_max_ = 0.001
3918 reflections	Δρ_max_ = 0.52 e Å^−^^3^
162 parameters	Δρ_min_ = −0.79 e Å^−^^3^
3 restraints	

### Special details

**Table:**

Geometry. All esds (except the esd in the dihedral angle between two l.s. planes) are estimated using the full covariance matrix. The cell esds are taken into account individually in the estimation of esds in distances, angles and torsion angles; correlations between esds in cell parameters are only used when they are defined by crystal symmetry. An approximate (isotropic) treatment of cell esds is used for estimating esds involving l.s. planes.

### Fractional atomic coordinates and isotropic or equivalent isotropic displacement parameters (Å^2^)

**Table:**

	*x*	*y*	*z*	*U*_iso_*/*U*_eq_	
Cs1	0.48526 (2)	0.71528 (3)	0.62552 (2)	0.04947 (8)	
O5	0.4180 (2)	0.7620 (5)	0.8390 (2)	0.0816 (9)	
H5A	0.371 (2)	0.806 (8)	0.869 (3)	0.122*	
H5B	0.4702 (17)	0.723 (7)	0.863 (3)	0.122*	
P1	0.66854 (5)	0.81111 (12)	0.39433 (6)	0.04547 (18)	
O1	0.67121 (16)	0.4475 (4)	0.50718 (17)	0.0667 (7)	
O2	0.57605 (15)	0.8484 (4)	0.43493 (16)	0.0618 (6)	
O3	0.65141 (15)	0.6826 (4)	0.29932 (16)	0.0597 (6)	
O4	0.70550 (16)	1.0147 (4)	0.3582 (2)	0.0768 (8)	
N1	0.75168 (18)	0.7207 (4)	0.45799 (19)	0.0499 (6)	
C1	0.74267 (19)	0.5511 (5)	0.50430 (18)	0.0438 (6)	
C2	0.82889 (19)	0.4816 (4)	0.55712 (17)	0.0415 (6)	
C3	0.8379 (3)	0.2855 (5)	0.5831 (2)	0.0534 (7)	
H3	0.789853	0.198137	0.569188	0.064*	
C4	0.9172 (3)	0.2191 (6)	0.6290 (3)	0.0668 (10)	
H4	0.922916	0.087133	0.645488	0.080*	
C5	0.9879 (2)	0.3473 (7)	0.6507 (3)	0.0682 (10)	
H5	1.041536	0.302436	0.682000	0.082*	
C6	0.9797 (2)	0.5411 (7)	0.6262 (2)	0.0607 (9)	
H6	1.027423	0.628199	0.641683	0.073*	
C7	0.90089 (19)	0.6085 (5)	0.5786 (2)	0.0477 (7)	
H7	0.896380	0.740050	0.561044	0.057*	
C8	0.7258 (3)	0.6063 (7)	0.2424 (3)	0.0770 (11)	
H8A	0.772305	0.550223	0.284658	0.116*	
H8B	0.752921	0.710446	0.205037	0.116*	
H8C	0.702224	0.506856	0.199145	0.116*	
C9	0.7984 (2)	1.0682 (6)	0.3429 (3)	0.0672 (9)	
H9A	0.815023	1.038514	0.276950	0.101*	
H9B	0.838067	0.996211	0.386767	0.101*	
H9C	0.805753	1.206393	0.354371	0.101*	

### Atomic displacement parameters (Å^2^)

**Table:**

	*U*^11^	*U*^22^	*U*^33^	*U*^12^	*U*^13^	*U*^23^
Cs1	0.05209 (12)	0.05443 (14)	0.04190 (11)	0.00312 (8)	0.00059 (8)	0.00041 (8)
O5	0.0608 (16)	0.129 (3)	0.0550 (15)	0.0128 (17)	0.0018 (13)	−0.0145 (15)
P1	0.0379 (4)	0.0511 (5)	0.0475 (4)	0.0007 (3)	0.0042 (3)	0.0067 (3)
O1	0.0604 (13)	0.0699 (17)	0.0694 (14)	−0.0275 (12)	−0.0167 (11)	0.0249 (12)
O2	0.0450 (11)	0.0797 (17)	0.0608 (13)	0.0057 (11)	0.0116 (10)	−0.0051 (12)
O3	0.0486 (12)	0.0814 (18)	0.0492 (12)	0.0121 (11)	0.0043 (10)	−0.0048 (11)
O4	0.0550 (13)	0.0640 (17)	0.111 (2)	−0.0022 (12)	−0.0041 (13)	0.0376 (15)
N1	0.0432 (13)	0.0525 (16)	0.0539 (14)	−0.0066 (11)	−0.0026 (11)	0.0116 (11)
C1	0.0461 (14)	0.0500 (17)	0.0353 (13)	−0.0080 (13)	0.0028 (11)	0.0029 (12)
C2	0.0455 (14)	0.0512 (17)	0.0279 (11)	−0.0044 (12)	0.0034 (10)	0.0001 (11)
C3	0.065 (2)	0.0528 (19)	0.0425 (15)	−0.0022 (15)	0.0017 (14)	0.0034 (13)
C4	0.079 (3)	0.063 (2)	0.058 (2)	0.0165 (19)	0.0020 (18)	0.0101 (17)
C5	0.057 (2)	0.096 (3)	0.0516 (18)	0.018 (2)	−0.0009 (15)	0.0060 (19)
C6	0.0455 (17)	0.087 (3)	0.0495 (17)	−0.0040 (16)	−0.0013 (13)	−0.0041 (16)
C7	0.0470 (15)	0.0526 (19)	0.0434 (14)	−0.0034 (14)	0.0025 (12)	−0.0033 (12)
C8	0.067 (2)	0.089 (3)	0.076 (2)	−0.001 (2)	0.0257 (19)	−0.020 (2)
C9	0.072 (2)	0.059 (2)	0.072 (2)	−0.0156 (18)	0.0157 (17)	0.0108 (17)

### Geometric parameters (Å, º)

**Table:**

Cs1—O1^i^	3.086 (2)	C1—C2	1.506 (4)
Cs1—O1	3.631 (3)	C2—C3	1.388 (4)
Cs1—O2^ii^	3.206 (3)	C2—C7	1.377 (4)
Cs1—O2	3.072 (2)	C3—H3	0.9300
Cs1—O3^iii^	3.431 (2)	C3—C4	1.374 (5)
Cs1—O3^i^	3.507 (3)	C4—H4	0.9300
Cs1—O4^ii^	3.310 (2)	C4—C5	1.369 (6)
Cs1—O5	3.112 (3)	C5—H5	0.9300
Cs1—O5^iv^	3.418 (4)	C5—C6	1.367 (6)
O5—H5A	0.853 (10)	C6—H6	0.9300
O5—H5B	0.856 (10)	C6—C7	1.379 (4)
P1—O2	1.468 (2)	C7—H7	0.9300
P1—O3	1.588 (2)	C8—H8A	0.9600
P1—O4	1.567 (3)	C8—H8B	0.9600
P1—N1	1.597 (3)	C8—H8C	0.9600
O1—C1	1.247 (3)	C9—H9A	0.9600
O3—C8	1.428 (4)	C9—H9B	0.9600
O4—C9	1.402 (4)	C9—H9C	0.9600
N1—C1	1.325 (4)		
			
O1^i^—Cs1—O5^iv^	93.03 (7)	C1—O1—Cs1^i^	141.26 (18)
O1^i^—Cs1—O5	111.22 (7)	C1—O1—Cs1	109.9 (2)
O1^i^—Cs1—O1	95.18 (5)	Cs1—O2—Cs1^ii^	112.10 (7)
O1^i^—Cs1—O2^ii^	89.03 (6)	P1—O2—Cs1	131.74 (14)
O1^i^—Cs1—O3^iii^	169.01 (6)	P1—O2—Cs1^ii^	108.03 (13)
O1^i^—Cs1—O3^i^	59.31 (5)	Cs1^vi^—O3—Cs1^i^	88.49 (5)
O1^i^—Cs1—O4^ii^	68.92 (7)	P1—O3—Cs1^i^	105.47 (10)
O2^ii^—Cs1—O5^iv^	169.77 (6)	P1—O3—Cs1^vi^	124.13 (11)
O2—Cs1—O5	155.59 (8)	C8—O3—Cs1^i^	107.7 (2)
O2—Cs1—O5^iv^	102.27 (6)	C8—O3—Cs1^vi^	102.3 (2)
O2^ii^—Cs1—O1	123.43 (5)	C8—O3—P1	122.6 (2)
O2—Cs1—O1	56.40 (6)	P1—O4—Cs1^ii^	100.77 (10)
O2—Cs1—O1^i^	85.16 (6)	C9—O4—Cs1^ii^	131.0 (2)
O2—Cs1—O2^ii^	67.90 (7)	C9—O4—P1	127.1 (2)
O2—Cs1—O3^iii^	103.57 (6)	C1—N1—P1	121.5 (2)
O2^ii^—Cs1—O3^iii^	100.36 (6)	O1—C1—N1	126.3 (3)
O2—Cs1—O3^i^	136.23 (6)	O1—C1—C2	118.8 (3)
O2^ii^—Cs1—O3^i^	129.70 (5)	N1—C1—C2	114.9 (2)
O2—Cs1—O4^ii^	104.60 (6)	C3—C2—C1	120.0 (3)
O2^ii^—Cs1—O4^ii^	43.69 (6)	C7—C2—C1	121.3 (3)
O3^i^—Cs1—O1	99.23 (5)	C7—C2—C3	118.7 (3)
O3^iii^—Cs1—O1	84.44 (5)	C2—C3—H3	119.7
O3^iii^—Cs1—O3^i^	109.86 (5)	C4—C3—C2	120.6 (3)
O4^ii^—Cs1—O5^iv^	145.89 (7)	C4—C3—H3	119.7
O4^ii^—Cs1—O1	157.27 (6)	C3—C4—H4	120.0
O4^ii^—Cs1—O3^iii^	114.31 (7)	C5—C4—C3	120.0 (4)
O4^ii^—Cs1—O3^i^	86.73 (6)	C5—C4—H4	120.0
O5—Cs1—O5^iv^	95.01 (7)	C4—C5—H5	120.0
O5^iv^—Cs1—O1	46.41 (6)	C6—C5—C4	119.9 (3)
O5—Cs1—O1	135.19 (7)	C6—C5—H5	120.0
O5—Cs1—O2^ii^	93.57 (7)	C5—C6—H6	119.8
O5^iv^—Cs1—O3^iii^	78.73 (7)	C5—C6—C7	120.4 (3)
O5—Cs1—O3^i^	67.78 (8)	C7—C6—H6	119.8
O5—Cs1—O3^iii^	62.88 (7)	C2—C7—C6	120.3 (3)
O5^iv^—Cs1—O3^i^	59.24 (6)	C2—C7—H7	119.9
O5—Cs1—O4^ii^	67.35 (7)	C6—C7—H7	119.9
Cs1—O5—Cs1^v^	95.57 (9)	O3—C8—H8A	109.5
Cs1—O5—H5A	138 (3)	O3—C8—H8B	109.5
Cs1^v^—O5—H5A	86 (4)	O3—C8—H8C	109.5
Cs1^v^—O5—H5B	82 (4)	H8A—C8—H8B	109.5
Cs1—O5—H5B	93 (3)	H8A—C8—H8C	109.5
H5A—O5—H5B	129 (3)	H8B—C8—H8C	109.5
O2—P1—O3	105.88 (13)	O4—C9—H9A	109.5
O2—P1—O4	106.13 (15)	O4—C9—H9B	109.5
O2—P1—N1	122.26 (14)	O4—C9—H9C	109.5
O3—P1—N1	110.27 (14)	H9A—C9—H9B	109.5
O4—P1—O3	106.15 (15)	H9A—C9—H9C	109.5
O4—P1—N1	105.09 (13)	H9B—C9—H9C	109.5
Cs1^i^—O1—Cs1	84.82 (5)		
			
Cs1—O1—C1—N1	−62.6 (3)	O4—P1—O3—Cs1^i^	−165.17 (10)
Cs1^i^—O1—C1—N1	44.7 (5)	O4—P1—O3—Cs1^vi^	−66.28 (16)
Cs1—O1—C1—C2	117.8 (2)	O4—P1—O3—C8	71.3 (3)
Cs1^i^—O1—C1—C2	−135.0 (3)	O4—P1—N1—C1	176.7 (2)
P1—N1—C1—O1	−2.4 (5)	N1—P1—O2—Cs1	−13.3 (3)
P1—N1—C1—C2	177.2 (2)	N1—P1—O2—Cs1^ii^	131.82 (14)
O1—C1—C2—C3	18.9 (4)	N1—P1—O3—Cs1^vi^	−179.60 (12)
O1—C1—C2—C7	−162.7 (3)	N1—P1—O3—Cs1^i^	81.52 (12)
O2—P1—O3—Cs1^i^	−52.64 (14)	N1—P1—O3—C8	−42.0 (3)
O2—P1—O3—Cs1^vi^	46.24 (18)	N1—P1—O4—Cs1^ii^	−141.69 (12)
O2—P1—O3—C8	−176.2 (3)	N1—P1—O4—C9	27.5 (4)
O2—P1—O4—Cs1^ii^	−10.89 (15)	N1—C1—C2—C3	−160.8 (3)
O2—P1—O4—C9	158.3 (3)	N1—C1—C2—C7	17.7 (4)
O2—P1—N1—C1	56.0 (3)	C1—C2—C3—C4	178.3 (3)
O3—P1—O2—Cs1	113.94 (18)	C1—C2—C7—C6	−179.3 (2)
O3—P1—O2—Cs1^ii^	−100.92 (13)	C2—C3—C4—C5	0.7 (5)
O3—P1—O4—Cs1^ii^	101.47 (12)	C3—C2—C7—C6	−0.8 (4)
O3—P1—O4—C9	−89.4 (3)	C3—C4—C5—C6	−0.2 (5)
O3—P1—N1—C1	−69.3 (3)	C4—C5—C6—C7	−0.8 (5)
O4—P1—O2—Cs1^ii^	11.62 (16)	C5—C6—C7—C2	1.3 (5)
O4—P1—O2—Cs1	−133.52 (18)	C7—C2—C3—C4	−0.2 (4)

Symmetry codes: (i) −*x*+1, −*y*+1, −*z*+1; (ii) −*x*+1, −*y*+2, −*z*+1; (iii) *x*, −*y*+3/2, *z*+1/2; (iv) −*x*+1, *y*−1/2, −*z*+3/2; (v) −*x*+1, *y*+1/2, −*z*+3/2; (vi) *x*, −*y*+3/2, *z*−1/2.

### Hydrogen-bond geometry (Å, º)

**Table:**

*D*—H···*A*	*D*—H	H···*A*	*D*···*A*	*D*—H···*A*
O5—H5*A*···O1^v^	0.85 (1)	2.05 (3)	2.785 (3)	143 (4)
O5—H5*B*···O2^iii^	0.86 (1)	1.87 (1)	2.721 (4)	170 (4)

Symmetry codes: (iii) *x*, −*y*+3/2, *z*+1/2; (v) −*x*+1, *y*+1/2, −*z*+3/2.
